# Convulsant Doses of a Dopamine D1 Receptor Agonist Result in Erk-Dependent Increases in Zif268 and Arc/Arg3.1 Expression in Mouse Dentate Gyrus

**DOI:** 10.1371/journal.pone.0019415

**Published:** 2011-05-03

**Authors:** Giuseppe Gangarossa, Manuela Di Benedetto, Gerard J. O'Sullivan, Mark Dunleavy, Cristina Alcacer, Alessandra Bonito-Oliva, David C. Henshall, John L. Waddington, Emmanuel Valjent, Gilberto Fisone

**Affiliations:** 1 Department of Neuroscience, Karolinska Institutet, Stockholm, Sweden; 2 Molecular and Cellular Therapeutics, Royal College of Surgeons in Ireland, Dublin, Ireland; 3 Department of Physiology and Medical Physics, Royal College of Surgeons in Ireland, Dublin, Ireland; 4 Institut National de la Santé et de la Recherche Médicale, UMR-S 839, Paris, France; 5 Université Pierre et Marie Curie, Paris, France; 6 Institut du Fer à Moulin, Paris, France; Institut National de la Santé et de la Recherche Médicale, France

## Abstract

Activation of dopamine D1 receptors (D1Rs) has been shown to induce epileptiform activity. We studied the molecular changes occurring in the hippocampus in response to the administration of the D1-type receptor agonist, SKF 81297. SKF 81297 at 2.5 and 5.0 mg/kg induced behavioural seizures. Electrophysiological recordings in the dentate gyrus revealed the presence of epileptiform discharges peaking at 30–45 min post-injection and declining by 60 min. Seizures were prevented by the D1-type receptor antagonist, SCH 23390, or the cannabinoid CB1 receptor agonist, CP 55,940. The effect of SKF 81297 was accompanied by increased phosphorylation of the extracellular signal-regulated protein kinases 1 and 2 (ERK), in the granule cells of the dentate gyrus. This effect was also observed in response to administration of other D1-type receptor agonists, such as SKF83822 and SKF83959. In addition, SKF 81297 increased the phosphorylation of the ribosomal protein S6 and histone H3, two downstream targets of ERK. These effects were prevented by genetic inactivation of D1Rs, or by pharmacological inhibition of ERK. SKF 81297 was also able to enhance the levels of Zif268 and Arc/Arg3.1, two immediate early genes involved in transcriptional regulation and synaptic plasticity. These changes may be involved in forms of activity-dependent plasticity linked to the manifestation of seizures and to the ability of dopamine to affect learning and memory.

## Introduction

Epileptic seizures are transient events due to abnormal or synchronous electrical activity in the brain. They are widely conceptualised to result from an imbalance between excitatory and inhibitory neurotransmission that affects various cerebral regions, such as the cerebral cortex, amygdala, thalamus and hippocampus. Among various models of prolonged seizures, those based on use of the cholinergic agonist pilocarpine or glutamate agonist kainic acid (KA) are well characterized; both induce epileptiform events that can develop into continuous seizures (status epilepticus; SE), leading to neuronal cell damage, particularly within the hippocampus [1], [2], [3]. In contrast, brief evoked or spontaneous seizures do not necessarily cause permanent cell loss [4]. The generation of epileptiform events can also be influenced by other neurotransmitter systems that enhance or decrease the threshold for seizure susceptibility. For instance, the endocannabinoid (eCB) system inhibits KA-induced seizures and promotes protective mechanisms through the activation of CB1 receptors in the hippocampus [5], [6].

Earlier studies indicate that dopamine lowers seizure threshold via activation of dopamine D1-type receptors, which include D1 (D1R) and D5 (D5R) receptors [7]. More recent evidence suggests that the ability of dopamine to induce seizures depends on activation of D1Rs coupled positively to cAMP signaling. Thus, seizures induced by administration of SKF 83822, a D1-type receptor agonist that selectively stimulates cAMP production, are blocked by deletion of D1Rs, but not of D5Rs, while SKF 83959, a D1-type receptor agonist that selectively stimulates phosphoinositide hydrolysis, fails to induce seizures [8]. Furthermore, SKF 83822-induced seizures are reduced by deletion of the dopamine- and cAMP-regulated phosphoprotein-32 kDa (DARPP-32) [9], a critical component of the cAMP signaling machinery implicated in D1R-mediated transmission [10]. However, the neural substrate(s) implicated in dopamine-induced seizures and the biochemical events associated with this phenomenon are still poorly understood.

In this study, we examined the ability of SKF 81297, a conventional D1-type receptor agonist, to generate epileptiform activity and affect signal transduction in the hippocampus. Our results indicate that systemic administration of SKF 81297 evokes acute seizures, which do not develop into SE or cause neuronal cell death, and that this effect is associated with a rapid and transient activation of the extracellular signal-regulated protein kinases 1 and 2 (ERK) pathway, specifically in the dentate gyrus (DG). By controlling nuclear and cytoplasmic downstream targets as well as a specific pattern of immediate early gene expression, the activation of the ERK pathway may promote some forms of activity-dependent plasticity in the dentate gyrus related to activation of D1Rs.

## Materials and Methods

### Mice

Male C57BL/6 mice were obtained at 8 weeks from Taconic (Tornbjerg, Denmark) and Harlan (Bicester, UK). Mutant mice with deletion of the *Drd1a* gene on an F2 hybrid (129×C57BL/6J) background were generated as described previously (Drago et al., 1994) and backcrossed to wildtype C57BL/6J for 5 generations. Analysis of isolated genomic DNA by PCR was used to genotype the progeny of heterozygous D1R mutant intermatings. Animals were housed in groups of five under standardized conditions with a 12 h light/dark cycle, stable temperature (20°C), and controlled humidity (40–50%). All studies were performed in accordance with the European Communities Council Directive 86/609/EEC for the care and use of experimental animals. These studies were approved by the Research Ethics Committee of Karolinska Institutet and the Royal College of Surgeons in Ireland. They were conducted under licence from the Swedish Animal Welfare Agency and the Department of Health & Children, Ireland, in accordance with the European Communities Council Directive 86/609/EEC for the care and use of experimental animals.

### Drugs

All drugs other than SL327 were purchased from Tocris (Bristol, UK) and injected i.p. in a volume of 10 ml/kg, for biochemical studies, or 4 ml/kg, for behavioural and electrophysiological studies. For biochemical studies, SKF 81297 and SCH 23390 were dissolved in 0.9% (w/v) saline. For electrophysiological studies SKF 81297 was dissolved in 0.5% DMSO and SCH 23390 in PCR-grade water. CP 55,940 and SL327 (α-[amino[(4-aminophenyl)-thio]-methylene]-2-(trifluoromethyl)-benzeneacetonitrile; an inhibitor of the mitogen-activated protein kinase/ERK kinase (MEK), which blocks the phosphorylation/activation of ERK), a gift from Dr. Edilio Borroni (Hoffmann-La Roche, Basel, Switzerland), were suspended by sonication in a solution of 8% Tween 80 in saline and administered i.p. 15 and 60 min before SKF 81297 injection, respectively. Control animals received equivalent volumes of the respective vehicles.

### Behavioural assessment

For evaluation of drug-induced seizures, mice were removed from their home cage and placed individually in clear glass observation cages (38×38×25 cm) with wood shavings as bedding material. Mice were then allowed to habituate for a period of 3 h to reduce initially high levels of activity in order to optimize detection of agonist-induced behavioural effects. Immediately following challenge with SKF 81297 (0.5–5.0 mg/kg) or vehicle, mice were recorded using digital video for 60 min. Video recordings was subsequently scored for seizures, characterized as any of the following phases occurring within the 60 min recording period: phase 1 = transient (≥5 sec) disruption of exploratory behaviour by tonic immobility/rigidity; phase 2 = rearing with forepaw myoclonus; phase 3 = generalized clonus; phase 4 = tonic-clonic seizure or rapid jumping and wild running (O'Sullivan et al. 2008); all recordings were scored by an observer who was unaware of the treatment of each animal. The D1R-type antagonist SCH 23390 (0.15 mg/kg) or the cannabinoid CB1 agonist CP 55,940 (0.25 mg/kg) were administered 5 and 15 min, respectively, prior to administration of SKF 81297. Mice were used once only.

### Electroencephalographic recording

Seizure studies were carried out in a manner similar to that described previously [9]. Briefly, for surgical implantation of extradural and intrahippocampal recording electrodes, mice were initially anaesthetised using 5% isoflurane (Abbott Laboratories, Kent, UK) in O_2_, placed in a stereotaxic frame and maintained under anaesthesia using 1.5% isoflurane in O_2_. Temperature was maintained normothermic (37±0.5°C) by means of a rectal thermometer and thermostatically controlled heating pad (Harvard Apparatus, MA, USA). The scalp was incised and a burr hole drilled in the exposed cranium over the hippocampus and the frontal cortex. A recording electrode and a frontal reference electrode (Plastics One Inc., VA, USA) were then implanted. A third complete craniotomy was drilled for the placement of a twisted bipolar electrode (Plastics One Inc., VA, USA) into the right dentate gyrus (coordinates from Bregma: AP = −2.0 mm, L = +0.5 mm, DV = −1.5 mm). All electrodes were affixed to the skull using dental cement (Kemdent, Wiltshire, UK). Following surgery, animals were placed in an incubator (Harvard Apparatus, MA, USA) to recover from anaesthesia before being returned to their home cage. Twenty-four hr post-surgery, experimental animals were connected to a digital EEG monitoring apparatus (AS40 amplifier system; Grass Technologies, RI, USA) and a 30 min baseline cortical/intrahippocampal EEG was recorded before administration of SKF 81297 (5.0 mg/kg) or vehicle. A subgroup of animals were pretreated with CP 55,940 (0.25 mg/kg) or vehicle, 30 min prior to SKF 81297. EEGs were recorded for 1 hr post-injection and subsequently analysed by a trained observer unaware of genotype or treatment for each animal. Electrographic seizures were defined as high frequency, high amplitude spiking (>1 Hz, more than twice baseline amplitude) with corresponding behavioural seizure activity.

### Tissue preparation and immunofluorescence

Procedures were as described previously [11], [12]. In brief, mice were rapidly anaesthetized by i.p. injection of pentobarbital (500 mg/kg, Sanofi-Aventis, France) prior to intracardiac perfusion of 4% (w/v) paraformaldehyde (PFA) in 0.1 M Na_2_HPO_4_/NaH_2_PO_4_ buffer, pH 7.5, delivered with a peristaltic pump at 20 ml/min over 5 min. Brains were post-fixed overnight in the same solution and stored at 4°C. Thirty µm-thick sections were cut with a vibratome (Leica, France) and stored at −20°C in a solution containing 30% (v/v) ethylene glycol, 30% (v/v) glycerol and 0.1 M sodium phosphate buffer, until they were processed for immunofluorescence. After permeabilization, free-floating sections were incubated with rabbit polyclonal antibodies for diphospho-ERK (phospho-Thr202/Tyr204-ERK1/2, 1∶500; Cell Signaling Technology, Beverly, MA, USA), phospho-Ser10-acetyl-Lys14-H3 (1∶500, Millipore, Stockholm, Sweden), phospho-Ser235/236-ribosomal protein S6 (rpS6; 1∶500, Cell Signaling Technology, Beverly, MA, USA) and mouse monoclonal antibody for MAP2 (1∶1000, Sigma-Aldrich, Sweden) overnight at 4°C. Antibodies for c-Fos (1∶400), Arc/Arg3.1 (1∶400), and Zif268 (1∶400) were purchased from Santa Cruz biotechnology and were incubated with tissue sections for 72 h at 4°C in a solution of BSA 1%. After three rinses in TBS, sections were incubated for 45 min at room temperature (RT) with goat-anti rabbit Cy3-coupled (1∶500) and goat-anti mouse Cy2-coupled (1∶500) secondary antibodies (Jackson ImmunoResearch Europe Ltd). A monoclonal antibody against VAMP2 directly coupled to Alexa 488 was used (1∶2000; gift from Dr. Peter Löw, Karolinska Institutet) [13]. Sections were then rinsed twice in TBS and twice in TB and mounted on a slide under cover slips using 1,4-diazabicyclo-[2. 2. 2]-octane (DABCO, Sigma-Aldrich Sweden AB, Stockholm). Fluoro-Jade® (Millipore Corp., Billica, MA, USA) staining was used to label degenerating neurons, as previously described [14].

### Analysis of immunofluorescence

Bilateral images were obtained using sequential laser scanning confocal microscopy (Zeiss LSM 510, Jena, Germany) and quantified using ImageJ software (National Institutes of Health). Quantification was performed in 325.75 µm×325.75 µm images by counting Cy2- and Cy3-immunofluorescent cells.

### Statistical analysis

Behavioural seizure events were summed over the 60 min recording period, expressed as means ± SEM and analysed using the Kruskal-Wallis non-parametric ANOVA, followed by Mann-Whitney U-test. Immunofluorescence data were expressed as means ± SEM and analyzed by one-way or two-way ANOVA followed by Bonferonni-Dunn test. Statistical analysis was performed using StatView software, with p<0.05 adopted as the threshold for significance.

## Results

### Seizure profile in response to systemic administration of SKF 81297

**Figure 1:**
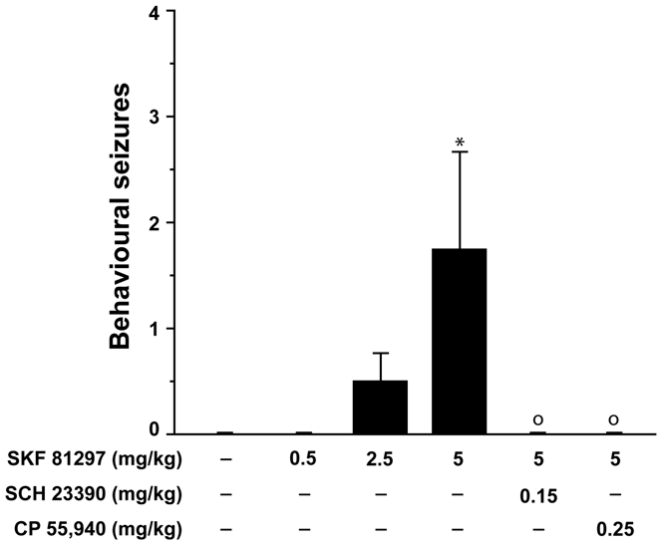
SKF 81297 induces behavioural seizures. Counts for seizure behaviours (any of phase 1–4 seizure phenotypes; see Materials and Methods) in mice treated with vehicle, SKF 81297 (0.5–5.0 mg/kg, i.p.) or SKF 81297 (5.0 mg/kg, i.p.) following administration of SCH 23390 (0.15 mg/kg, i.p.) or CP 55,940 (0.25 mg/kg, i.p.). Data (means ± SEM; n = 8 mice per group) were analysed using Kruskal-Wallis nonparametric ANOVA: dose-response for induction of seizures by SKF 81297 (p<0.05); Mann-Whitney U-tests: * p<0.05 vehicle vs. SKF 81297; ° p<0.05 SKF 81297 vs. SKF 81297 following pre-treatment with SCH 23390 and SKF 81297 following pre-treatment with CP 55,940.

SKF 81297 (0.5–5.0 mg/kg) dose-dependently induced behavioural seizures (p<0.05; Fig. 1), which were observed in 6 (75%) of 8 mice receiving 5.0 mg/kg. Among the 4 phases of seizure activity recorded (see materials and methods), there was no significant preponderance of any seizure phenotype. Behavioural seizures induced by 5.0 mg/kg SKF 81297 were abolished by pretreatment with either 0.15 mg/kg SCH 23390 or 0.25 mg/kg CP 55,940 (each p<0.05; Fig. 1).

**Figure 2:**
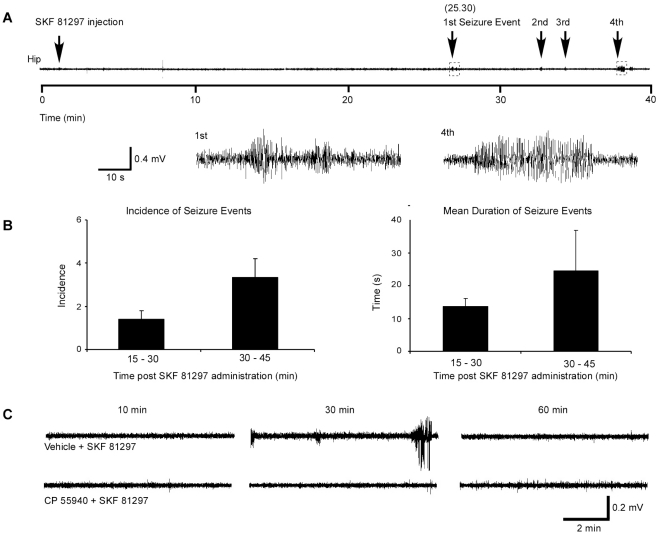
SKF 81297 induces electrophysiological seizures. (A) Representative trace showing hippocampal electrographic seizure events during 60 min following administration of SKF 81297 (5.0 mg/kg, i.p.). Lower panel shows in detail the first and fourth electrographic events. (B) Quantification of seizure incidence and mean seizure duration determined over a 60 min period of observation. Mean ± SEM (n = 5 animals). Seizure incidence and duration peaked between 30–45 min. Electrographic events between 0–15 min and 45–60 min were observed in only one animal each (data not shown). (C) Representative hippocampal traces over 60 min recording period showing seizures following SKF 81297 administration. Pretreatment with CP 55,940 (0.25 mg/kg, i.p.) abolishes all SKF 81297-induced seizures.

Simultaneous EEG recordings from skull-mounted surface electrodes and intra-cerebral depth recordings from the DG following injection of SKF 81297 (5.0 mg/kg) demonstrated the presence of seizures (high amplitude and high frequency discharges) in the DG in 5 (71%) of 7 mice; mean latency to emergence of seizures was 22±2 min. There was a time-dependent increase in seizure incidence and duration following injection of SKF 81297 (Fig. 2), with seizure severity peaking at 30–45 min post-injection and declining by 60 min (Fig. 2B, C). No seizure activity was evident in vehicle-treated mice (n = 5; data not shown).

The SKF 81297-induced seizures were not accompanied by the appearance of neurodegenerative responses, as determined by Fluoro-Jade staining, 6 hrs after recording (data not shown).

### SKF 81297-induced seizures are associated with an increase of ERK phosphorylation selectively in the dentate gyrus of the hippocampus

**Figure 3:**
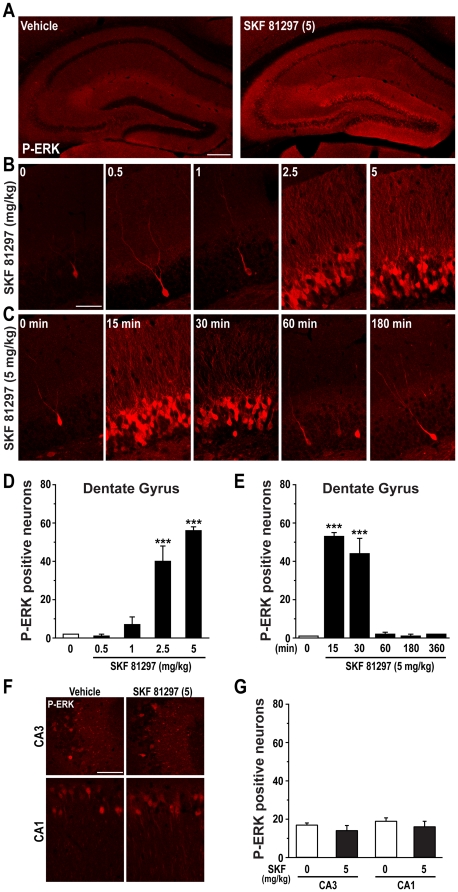
SKF 81297 induces ERK phosphorylation in the dentate gyrus. (A) Single confocal section showing phosphorylated ERK (P-ERK) in the hippocampus of mice treated with vehicle or SKF 81297 (5.0 mg/kg, i.p.). Scale bar: 150 µm. (B) Dose-response (0, 0.5, 1, 2.5, and 5.0 mg/kg) and (C) time course (0, 15, 30, 60 and 180 min) of P-ERK in mice treated with SKF 81297. Scale bar: 40 µm. (D and E) Quantification of phosphorylated ERK (P-ERK) immunoreactive neurons in the dentate gyrus of mice treated with vehicle or SKF 81297. Data (means ± SEM; n = 3–4 mice per group) were analyzed using one-way ANOVA: dose-response (F_(4,9)_ = 61.22; p<0.001), time course (F_(5,12)_ = 239.78; p<0.001). (F) Single confocal section showing P-ERK in the CA3 and CA1 areas of mice treated with vehicle or SKF 81297 (5.0 mg/kg, i.p.). Scale bar: 40 µm. (G) Quantification of P-ERK immunoreactive neurons in CA3 and CA1 of mice treated with vehicle or SKF 81297. Data (means ± SEM; n = 4–5 mice per group) were analyzed using student t-test: CA3 (F_(1,7)_ = 1.23; NS), CA1 (F_(1,7)_ = 0.65; NS).

**Figure 4:**
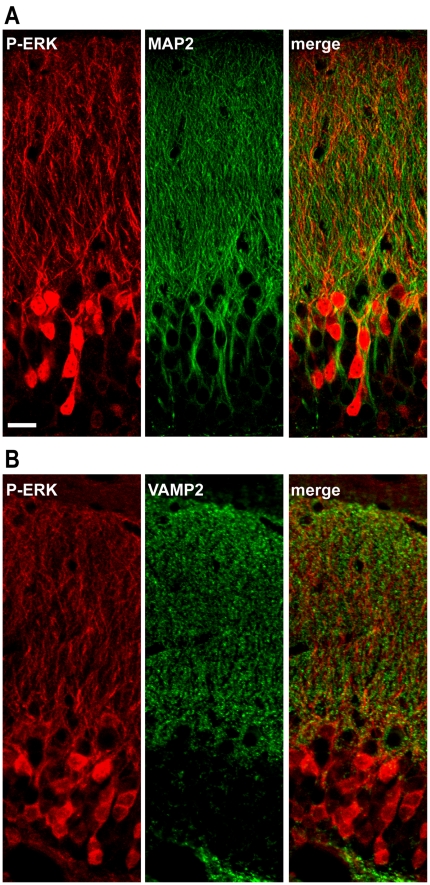
SKF 81297-induced ERK phosphorylation occurs at the postsynaptic level. (A and B) Single confocal sections showing P-ERK) (red) and MAP2 (green, upper panel) or VAMP2 (green, lower panel) immunofluorescence in the dentate gyrus of mice 15 min after SKF 81297 (5.0 mg/kg, i.p.). Note that P-ERK immunoreactive neurons are observed in MAP2-positive cells, suggesting that ERK activation induced by SKF 81297 occurred mainly at the postsynaptic level. Scale bar: 20 µm.

Pharmacological induction of seizures by KA and pilocarpine is accompanied by increased ERK phosphorylation in several hippocampal regions [15], [16], [17]. Therefore we examined the induction and distribution of phospho-ERK (P-ERK) in the hippocampus of mice treated with SKF 81297. Administration of 2.5 and 5.0 mg/kg SKF 81297 resulted in a large increase in P-ERK immunoreactivity selectively in the granule cell layer of the DG (Fig. 3A, B and D). This effect peaked at 15–30 min post-injection and declined by 60 min (Fig. 3C and E). P-ERK immunoreactivity was detected in neuronal nuclei and perikarya, as well as in the dendrites of almost all the granule cells of the DG (Fig. 3A–C). In contrast, administration of 5.0 mg/kg SKF 81297 did not affect ERK phosphorylation in the CA3 and CA1 pyramidal neurons (Fig. 3F and G). Dendritic activation of ERK was confirmed by the strong co-localization of P-ERK with the microtubule-associated protein 2 (MAP2), a protein highly enriched in dendrites (Fig. 4A). In contrast, no co-localization of P-ERK immunoreactivity was found with a marker of nerve terminals, the vesicle-associated membrane protein 2 (synaptobrevin 2, also known as VAMP2) (Fig. 4B). The effect of SKF 81297 (5 mg/kg) on ERK phosphorylation was compared to those produced by the D1-type agonists SKF 83822 and SKF 83959, which are considered to preferentially activate cAMP and phospholipase C signaling, respectively [9]. We found that SKF 83822 (2 mg/kg) produced an increase in the number of P-ERK positive neurons comparable to that of SKF 81297 (Figure S1). The same dose of SKF 83959 was also able to increase ERK phosphorylation, however its effect was significantly lower than those produced by SKF 81297 or SKF 83822 and appeared to be preferentially exerted in the cytoplasm (Figure S1).

### SKF 81297-induced ERK phosphorylation is prevented by inactivation of dopamine D1 receptors

**Figure 5:**
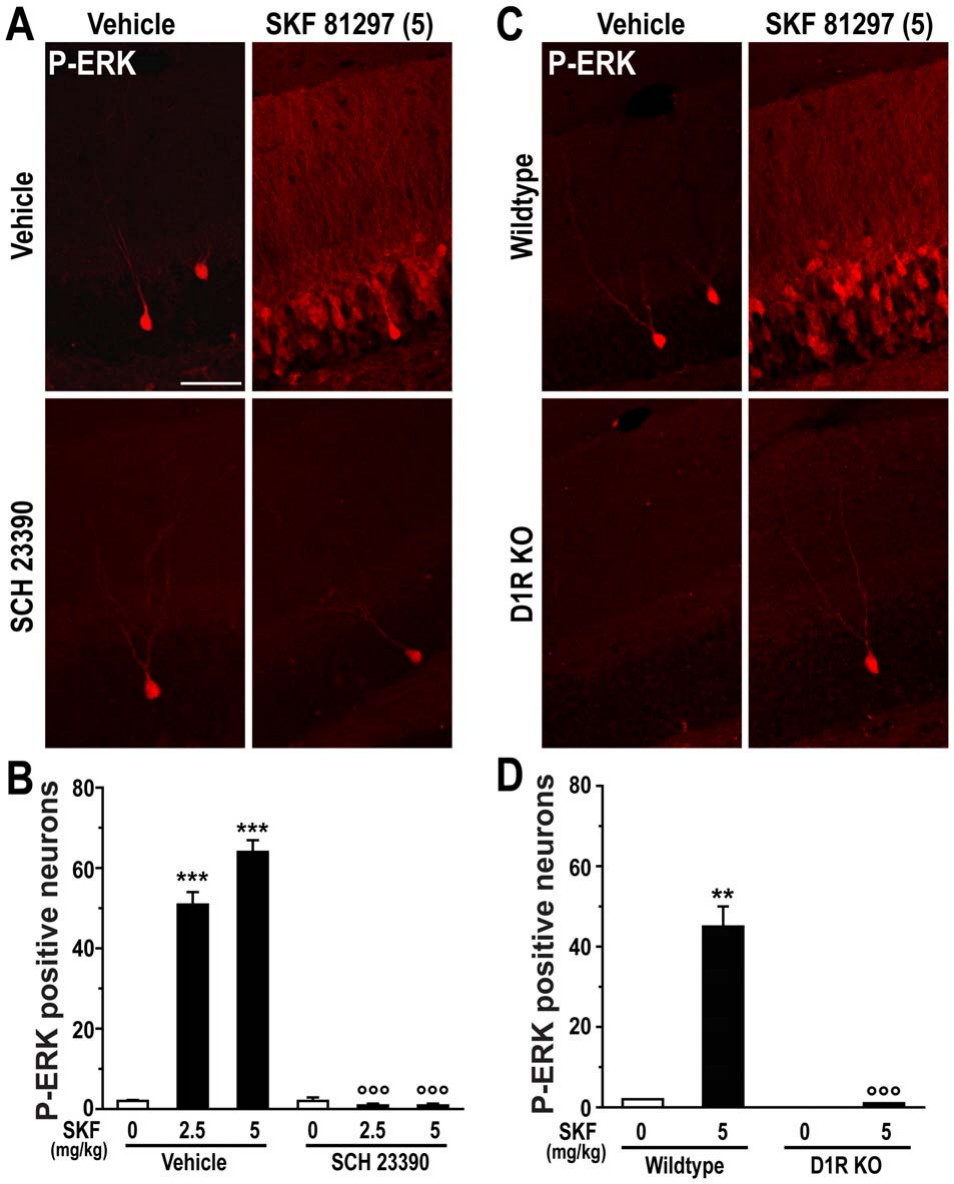
D1Rs are required for SKF 81297-induced ERK phosphorylation in the dentate gyrus. (A) Immunohistochemical detection of P-ERK in the granule cell layer of mice treated with vehicle, SKF 81297 (SKF; 5.0 mg/kg, i.p.) alone or in combination with SCH 23390 (0.15 mg/kg, i.p.). Scale bar: 40 µm. (B) Quantification of P-ERK immunoreactive neurons in the dentate gyrus of mice treated with vehicle and SKF 81297 alone or in combination with SCH 23390 (0.15 mg/kg, i.p.). Data (means ± SEM; n = 2–4 mice per group) were analyzed using two-way ANOVA: effect of SKF 81297 (F_(2,11)_ = 77.09; p<0.001), SCH 23390 (F_(1,11)_ = 347.53; p<0.001), interaction (F_(2,11)_ = 77.97; p<0.01). (C) Immunohistochemical detection of P-ERK in the granule cell layer of wildtype or D1R knock out (D1R KO) mice treated with vehicle or SKF 81297. Scale bar: 40 µm. (D) Quantification of P-ERK immunoreactive neurons in the dentate gyrus of wildtype or D1R knock out (D1R KO) mice treated with vehicle or SKF 81297. Data (means ± SEM; n = 2–3 mice per group) were analyzed using two-way ANOVA: effect of genotype (F_(1,6)_ = 48.21; p<0.001), treatment (F_(1,6)_ = 44.11; p<0.001, interaction (F_(1,6)_ = 40.19; p<0.001). Bonferroni-Dunn test: ** p<0.01, *** p<0.001 vehicle vs. SKF 81297; °°° p<0.001 vehicle vs. SCH 23390 and wildtype vs. D1R KO.

The increase in ERK phosphorylation produced by 2.5 or 5.0 mg/kg SKF 81297 was abolished by 0.15 mg/kg SCH 23390 (Fig. 5A and B) and was absent in mice with deletion of D1Rs (Fig. 5C and D). These results indicate that SKF 81297-induced seizures increase ERK phosphorylation in the DG through stimulation of D1Rs.

### SKF 81297 induces phospho-acetylation of histone H3 and phosphorylation of rpS6

**Figure 6:**
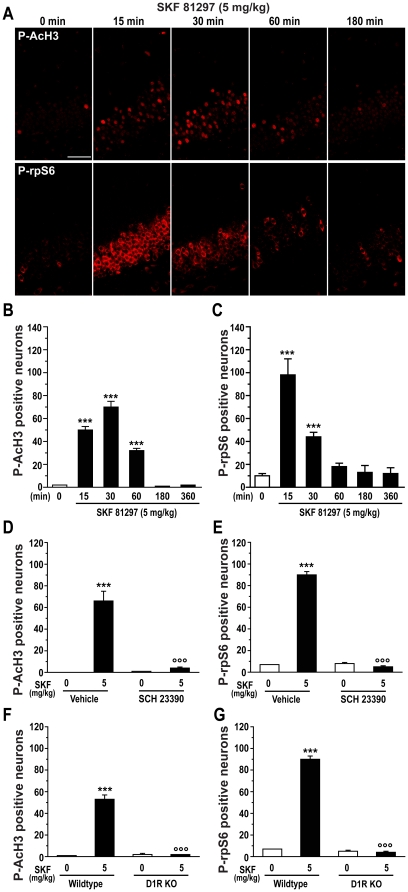
SKF 81297 induces histone H3 phospho-acetylation and rpS6 phosphorylation in the dentate gyrus. (A) Single confocal sections showing immunofluorescence detection of phospho-Ser10-acetyl-Lys14 histone H3 (P-AcH3, upper panel) and phospho-rpS6 (P-rpS6, lower panel) in mice treated with vehicle or SKF 81297 (5.0 mg/kg, i.p.). Scale bar: 40 µm. (B and C) Quantification of P-AcH3 and P-rpS6 immunoreactive neurons in time course study performed in mice treated with vehicle or SKF 81297 (5.0 mg/kg, i.p.). Data (means ± SEM; n = 3 mice per group) were analyzed using one-way ANOVA: P-AcH3 (F_(5,12)_ = 148.78; p<0.001); P-rpS6 (F_(5,20)_ = 33.45; p<0.001). (D and E) Quantification of P-AcH3 and P-rpS6 immunoreactive neurons after pharmacological antagonism with the D1-like family antagonist SCH 23390 (0.15 mg/kg, i.p.). Data (means ± SEM; n = 4 mice per group) were analyzed using two-way ANOVA: P-AcH3, effect of SKF 81297 (F_(1,9)_ = 67.38; p<0.001), SCH 23390 (F_(1,9)_ = 55.25; p<0.001), interaction (F_(1,9)_ = 57.04; p<0.001); P-rpS6, effect of SKF 81297 (F_(1,7)_ = 352.36; p<0.001), SCH 23390 (F_(1,7)_ = 383.77; p<0.001), interaction (F_(1,7)_ = 402.32; p<0.001). (F and G) Quantification of P-AcH3 and P-rpS6 immunoreactive neurons in wildtype and D1R knock out (D1R KO) mice treated with vehicle or SKF 81297 (5.0 mg/kg, i.p.). Data (means ± SEM; n = 2–4 mice per group) were analyzed using two-way ANOVA: P-AcH3, effect of genotype (F_(1,8)_ = 56.01; p<0.001), treatment (F_(1,8)_ = 59.37; p<0.001), interaction (F_(1,8)_ = 58.24; p<0.001); P-rpS6, effect of genotype (F_(1,6)_ = 360.91; p<0.001), treatment (F_(1,6)_ = 313.63; p<0.001), interaction (F_(1,6)_ = 321.28; p<0.001). Bonferroni-Dunn test: *** p<0.001 vehicle vs. SKF 81297, °°° p<0.001 vehicle vs. SCH 23390 or wildtype vs. D1R KO.

We proceeded by determining whether SKF 81297-induced seizures enhanced phosphorylation of histone H3, an important downstream nuclear target of ERK involved in the nucleosomal response [18]. Mice given 5.0 mg/kg SKF 81297 showed a robust increase in the number of phospho-acetyl-H3 (P-AcH3) immunoreactive neurons, restricted to the granule cell layer of the DG (Fig. 6A, upper panels and B); P-AcH3 was maximal 30 min after SKF 81297 administration and returned to basal levels within 3 hr (Fig. 6A and B). This effect of SKF 81297 was abolished by 0.15 mg/kg SCH 23390 (Fig. 6D) and by deletion of D1Rs (Fig. 6F).

We next examined the state of phosphorylation of rpS6, a component of the 40S ribosomal subunit [19] which can be phosphorylated by ERK via the 90 kDa ribosomal S6 kinases (RSK) at the dual site Ser235/236 [20]. Mice given 5.0 mg/kg SKF 81297 showed a rapid, transient increase in the number of phospho-S6 immunoreactive neurons selectively in the granule cells of the DG (Fig. 6A, lower panel and C). This effect of SKF 81297 was abolished by 0.15 mg/kg SCH 23390 (Fig. 6E) and by deletion of D1Rs (Fig. 6G).

### Activation of CB1 receptors abolishes SKF 81297-induced seizures and reduces phosphorylation of ERK, AcH3 and rpS6

**Figure 7:**
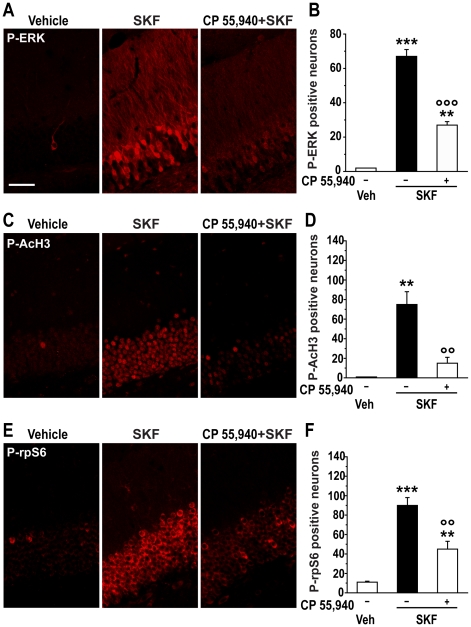
Effect of CB1 receptor activation on ERK, AcH3 and rpS6 phosphorylation. Immunohistochemical detection of P-ERK (A), P-AcH3 (C) and P-rpS6 (E) in the granule cell layer of mice treated with vehicle, SKF 81297 (SKF; 5.0 mg/kg) alone or in combination with the CB1 receptor agonist, CP 55,940 (0.25 mg/kg, i.p.). Scale bar: 40 µm. (B, D and F) Quantification of P-ERK, P-AcH3 and P-rpS6 immunoreactivity in mice treated with vehicle, SKF alone or in combination with CP 55,940. Data (means ± SEM; n = 2–4 mice per group) were analyzed using one-way ANOVA: P-ERK (F_(2,5)_ = 149.01, p<0.001); P-AcH3 (F_(2,6)_ = 13.79, p<0.01); P-rpS6 (F_(2,9)_ = 30.08, p<0.001). Bonferroni-Dunn test: ** p<0.01, *** p<0.001 vehicle vs. SKF, °° p<0.01, °°° p<0.001 vs. SKF.

Activation of CB1 receptors, which are abundantly expressed in the hippocampus, has been shown to reduce KA-induced seizures [5], [6]. CP 55,940 (0.25 mg/kg) antagonized SKF 81297 (5.0 mg/kg)-induced phosphorylation of ERK (Fig. 7A and B), AcH3 (Fig. 7C and D) and rpS6 (Fig. 7E and F) in the granular cells of the DG. Thus endocannabinoids, through activation of CB1 receptors, appear to exert an anticonvulsant effect against D1R-induced seizures.

### SKF 81297-induced phosphorylation of AcH3 and rpS6 depends on ERK activation

**Figure 8:**
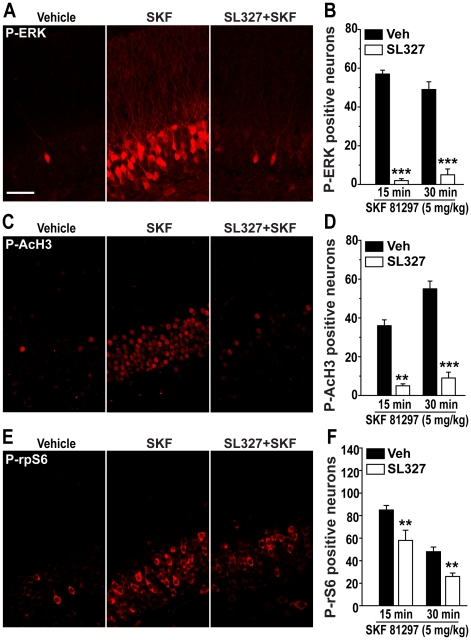
AcH3 and rpS6 phosphorylation induced by SKF 81297 is dependent on ERK activation. Immunohistochemical detection of P-ERK (A), P-AcH3 (C) and P-rpS6 (E) in the granule cell layer of mice treated with vehicle, SKF 81297 (5.0 mg/kg, i.p.) alone or in combination with the MEK inhibitor, SL327 (50 mg/kg, i.p.). Scale bar: 40 µm. (B, D and F) Quantification of P-ERK, P-AcH3 and P-rpS6 immunoreactive neurons in mice treated with SKF 81297 alone or in combination with SL327 (50 mg/kg, i.p.) and perfused 15 and 30 min later. Data (means ± SEM; n = 4–5 mice per group) were analyzed using two-way ANOVA: P-ERK, effect of time (F_(1,10)_ = 0.61, NS), treatment (F_(1,10)_ = 235.19, p<0.001), interaction (F_(1,10)_ = 2.91, NS); P-AcH3, effect of time (F_(1,10)_ = 8.05, p<0.02), treatment (F_(1,10)_ = 91.73, p<0.001), interaction F_(1,10)_ = 4.27, NS); P-rpS6, effect of time (F_(1,17)_ = 34.54, p<0.001), treatment (F_(1,17)_ = 17.67, p<0.001), interaction (F_(1,17)_ = 0.16, NS). Bonferroni-Dunn test: ** p<0.01, *** p<0.001 vehicle vs. SL327.

Parallel increases in the state of phosphorylation of ERK, histone H3 and rpS6 produced by administration of SKF 81297 suggest a causal link between these effects. To examine this possibility we used SL237, an inhibitor of mitogen-activated protein kinase/ERK kinase (MEK) which blocks the phosphorylation/activation of ERK. The increase in ERK phosphorylation induced by 5.0 mg/kg SKF 81297 was antagonized by a 60 min pretreatment with 50 mg/kg SL327 (Fig. 8A and B). The blockade of ERK phosphorylation was accompanied by antagonism of phosphorylation of AcH3 (Fig. 8C and D) and P-rpS6 (Fig. 8E and F).

### SKF 81297 promotes the expression of Arc/Arg3.1 and Zif268 via activation of ERK

**Figure 9:**
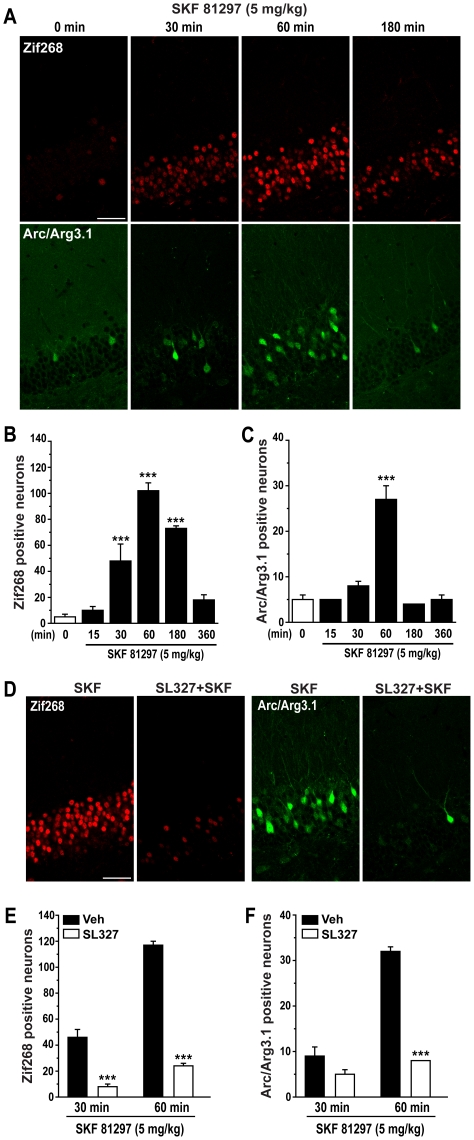
Zif268 and Arc/Arg3.1 expression induced by SKF 81297 is dependent on ERK activation. (A) Single confocal sections showing immunofluorescence detection of Zif268 and Arc/Arg3.1 protein expression at various times after SKF 81297 (5.0 mg/kg, i.p.) injection. Scale bar: 40 µm. (B and C) Quantification of Zif268 and Arc/Arg3.1 immunoreactive neurons in mice treated with vehicle or SKF 81297 (5.0 mg/kg, i.p.) at various time points. Data (means ± SEM; n = 3–6 mice per group) were analyzed using one-way ANOVA: Zif268 (F_(5,12)_ = 36.50; p<0.001), Arc/Arg3.1 (F_(5,21)_ = 23.50; p<0.001). (D) Immunohistochemical detection of Zif268 and Arc/Arg3.1 and protein expression in the granule cell layer of mice treated with SKF 81297 (5.0 mg/kg) alone or in combination with SL327 (50 mg/kg, i.p.). Scale bar: 40 µm. (E and F) Quantification of Zif268 and Arc/Arg3.1 immunoreactive neurons in mice treated with SKF 81297 (5.0 mg/kg) alone or in combination with SL327 (50 mg/kg, i.p.) at two time points. Data (means ± SEM; n = 3–4 mice per group) were analyzed using two-way ANOVA: Zif268, effect of time (F_(1,10)_ = 105.87, p<0.001), SL327 (F_(1,10)_ = 239.57, p<0.001), interaction (F_(1,10)_ = 42.45, p<0.001); Arc/Arg3.1, effect of time (F_(1,12)_ = 123.88, p<0.001), SL327 (F_(1,12)_ = 143.47, p<0.001), interaction (F_(1,12)_ = 70.12, p<0.001). Bonferroni-Dunn test: *** p<0.001 vehicle vs. SL327.

We next investigated whether systemic activation of D1Rs is accompanied by induction of immediate early genes (IEGs) in the hippocampus by determining the effect of 5.0 mg/kg SKF 81297 on levels of Arc/Arg3.1, Zif268 and c-Fos. SKF 81297 induced a large increase in expression of Zif268 and Arc/Arg3.1, specifically in the granule cell layer of the DG (Fig. 9A); Zif268 expression increased at 30 min, peaked at 60 min and returned to basal levels 360 min after SKF 81297 administration (Fig. 9B). The increase in Arc/Arg3.1 expression was more transient; Arc/Arg3.1 expression was first observed at 60 min and returned to basal level 180 min after SKF 81297 administration (Fig. 9C). Increases in Arc/Arg3.1 and Zif268 expression induced by SKF 81297 (5.0 mg/kg) were abolished by pre-treatment with 50 mg/kg SL327 (Fig. 9D, E and F). In contrast, SKF 81297 administration induced only a very modest increase in c-Fos expression in dentate gyrus which did not reach statistical significance when using the Bonferroni post-hoc test (Figure S2).

## Discussion

This study describes a series of unique molecular changes occurring in the hippocampal formation in association with the emergence of epileptiform activity induced by activation of D1Rs. These changes are distinct from those produced by acute injection of chemoconvulsants, such as pilocarpine or KA, and involve increased activation of ERK specifically in the cytoplasm and nuclei of granule cells, leading to phosphorylation of histone H3 and rpS6, and enhanced expression of Zif268 and Arc/Arg3.1.

It has been shown that D1R agonists synergise with subthreshold doses of pilocarpine to induce SE [7]. In addition, it has been reported that, in the mouse, administration of the D1-type receptor agonist SKF 83822 generates seizures [8], [9]. This pro-convulsant effect requires intact D1Rs, but not D5Rs, and involves activation of cAMP signaling. In addition, western blot analysis revealed that SKF 83822-induced epileptiform activity was paralleled by a large increase in phospho-ERK immunoreactivity in the hippocampus [9].

Increased ERK phosphorylation in the hippocampal formation is a common response induced by chemoconvulsants. Administration of pilocarpine at doses able to induce SE and spontaneous seizures increases ERK phosphorylation in the DG, including the hilar region, and, to a lesser extent, in the CA1 [16], [21]. In this study, we show that administration of the D1-type receptor agonist SKF 81297 is also able to enhance phospho-ERK immunoreactivity in the DG. However, we do not observe any increase in ERK phosphorylation in the CA3 and CA1 pyramidal neurons. Moreover, SKF 81297 does not increase ERK phosphorylation in the interneurons of the hilar region that are affected by administration of pilocarpine and KA [16], [22]. Thus, it appears that the degree of activation produced by SKF 81297 in the DG is lower than that necessary to model temporal lobe epilepsy. This is in line with the inability of SKF 81297 to generate SE, neuronal damage or later spontaneous seizures.

The increase in ERK phosphorylation produced by SKF 81297 is abolished by genetic inactivation of D1Rs, which also prevents the ability of another D1-type receptor agonist, SKF 83822, to induce epileptiform activity [8], [9]. The existence of a link between D1R-mediated induction of seizures and ERK signaling is further suggested by the observation that administration of the D1-type receptor antagonist SCH 23390, or the cannabinoid CB1 agonist CP 55,940, which prevent the manifestation of epileptiform activity, also prevent the ability of SKF 81297 to activate ERK.

The increase in ERK phosphorylation produced in the DG by administration of SKF 81297 is short-lived and disappears within 60 min after drug administration. This contrasts with the effects of pilocarpine and KA, whose administration leads to increases in ERK phosphorylation that last for hours [16], [17], [21]. Overall, the transient activation of ERK induced by SKF 81297 corresponds to the number and duration of seizure events, which peak at 30 min following SKF 81297 administration and decline by 60 min. A more precise analysis of the time at which the first seizures are generated suggests that maximal ERK phosphorylation, which occurs within 15 min after drug administration, may precede the onset of epileptiform activity, which occurs at 20–25 min after drug administration. However that ERK plays a role in the generation of seizures is challenged by the observation that administration of SL327, which abolishes ERK phosphorylation, does not affect pilocarpine-induced seizures [21]. Therefore, increased ERK signaling in the DG most likely represents a marker of neuronal activation indicative of D1R agonist-induced seizures, rather than the cause of such seizures. Interestingly, recent work in a mouse model of pilocarpine-induced temporal lobe epilepsy proposes that phosphorylated ERK may represent an early indicator of activated neurons during spontaneous seizures [23]. Furthermore, the spatio-temporal patterns of ERK phosphorylation associated with D1R-mediated seizures and spontaneous (i.e. epileptic) seizures are very similar, suggesting that these two phenomena may recruit the same hippocampal circuits.

The activation of ERK produced by administration of SKF 81297 occurs both in the cytoplasm and in the nucleus of granule cells, as shown by the presence of phosphorylated ERK in these two compartments. In line with this observation, we found that activation of D1Rs increased the state of phosphorylation of rpS6 and histone H3, two downstream targets of ERK selectively expressed in the cytoplasm and the nucleus, respectively. Time-course analysis indicated a rapid increase in rpS6 phosphorylation, which peaked at 15 min after administration of SKF 81297, and a more progressive increase in histone H3 phosphorylation, which reached a peak at 30 min and was still significantly elevated at 60 min. Such kinetics of phosphorylation indicates a rapid and more transient activation of ERK signaling in the cytoplasm, paralleled by a slower and more resilient activation in the nucleus.

Phosphorylation of rpS6 is likely to occur via activation of RSK, a direct ERK substrate and major rpS6 kinase, phosphorylation of which was also enhanced following SKF 81297 administration (data not shown). However, the increase in rpS6 phosphorylation produced by SKF 81297 was only partially reduced by SL327, a drug, which prevents the phosphorylation of ERK, indicating the involvement of other signaling cascades in D1R-mediated control of this particular downstream target. In contrast, SKF 81297-induced phosphorylation of histone H3 at Ser10 was abolished by pretreatment with SL327. Surprisingly, ERK activation was not accompanied by increased phosphorylation of MSK1 or MSK2 (data not shown), two major substrates of ERK critically involved in histone H3 phosphorylation [24], [25], [26]. Therefore, the molecular link between ERK activation and histone H3 phosphorylation remains to be identified.

Induction of gene expression has been extensively studied in various experimental models of seizures. For instance, increased expression of genes coding for activity-regulated transcription factors, such as *c-fos* and *zif268*, has been demonstrated in the hippocampus after kindling [27], [28], or following electrical or chemical induction of seizures [29], [30], [31], [32], [33], [34]. Moreover a strong induction of c-Fos has been observed in the granule neurons of the DG following a spontaneous seizure [23].

In the present study, we found a large increase in levels of Zif268 in the nuclei of granule cells, which followed temporally the increase in ERK and histone H3 phosphorylation produced by SKF 81297. This effect was prevented by treatment with SL327, indicating that phosphorylation of ERK and, possibly, of histone H3 is implicated in the expression of this gene. However, previous studies showed that inhibition of MSK1, which prevents cocaine-induced phosphorylation of histone H3 at Ser10, did not prevent the ability of this drug to increase the expression of zif268 [24]. Thus, further studies will be necessary in order to demonstrate the involvement of histone H3 phosphorylation in the regulation of zif268 expression, in the dentate gyrus.

In contrast to Zif268, we found only a small increase in c-Fos expression following D1R activation in the dentate gyrus. The pattern of immediate early gene induction observed with SKF 81297 is similar to that described following high frequency stimulation of the perforant pathway, which induces LTP in the DG. Thus, tetanising stimuli increase Zif268, without consistently affecting c-Fos expression [35], [36], [37], [38]. Moreover, the increase in Zif268 expression associated with LTP is prevented by inhibition of ERK with SL327 [39].

Administration of SKF 81297 increases also the levels of *Arc/Arg3.1*, another gene involved in multiple forms of neuronal plasticity, including LTP and LTD. Previous work proposed that the involvement of Zif-268 in the late phase of LTP is related to its ability to promote *Arc/Arg3.1* transcription [40]. Levels of Arc/Arg3.1 are also regulated at the translational level, following rapid transport of mRNA to dendrites [41]. Thus, the increased expression of *Arc/Arg3.1* induced by activation of D1Rs may depend on concomitant ERK-dependent phosphorylation of histone H3, which may be implicated in the nucleosomal response, and rpS6, which may instead regulate local synaptic translation of mRNA encoded by *Arc/Arg3.1*. In support of this possibility, it has recently been shown that, in the hippocampus, activation of ERK promotes local Arc/Arg3.1 expression independently of other signaling pathways involved in translational control, such as the mammalian target of the rapamycin cascade [42].

Changes in the expression of *zif268* and *Arc/Arg3.1* reported here have been studied with respect to their involvement in hippocampal plasticity. Dopamine transmission has been implicated in various forms of learning and memory, in which the hippocampal formation is though to play an essential role. In many cases, the action of dopamine has been proposed to occur via activation of D1- or D2-type receptors located in various anatomical regions within the hippocampal formation. It will be interesting to define whether the effects described here are produced by a direct action of SKF 81297 on the granule cells of the DG, or by an indirect mechanism involving a polysynaptic circuit.

In conclusion, the present study indicates that the epileptiform activity produced by systemic administration of a D1R agonist is associated with activation of ERK in the granule cells of the DG. This effect is accompanied, in the nucleus, by increased phosphorylation of histone H3 and expression of *zif268*. Moreover, administration of SKF 81297 enhances phosphorylation of rpS6 in the cytoplasm and promotes the expression of Arc/Arg3.1. These changes are distinct from those produced by chemoconvulsants, such as pilocarpine and KA, which cause a much more pervasive and prolonged activation of the DG and other hippocampal regions and which trigger the expression of additional immediate early genes. The more restricted action of SKF 81297 is consistent with the inability of this drug to induce SE, spontaneous seizures and neuronal cell death, and further supports the notion that the DG acts as a gate and sensor able to detect a wide range of stimuli impinging on the hippocampus. Future studies will be required to elucidate the significance of D1R-mediated molecular changes in hippocampal plasticity and function.

## Supporting Information

Figure S1 **Effect of SKF 81297 on c-Fos expression in the dentate gyrus.** (A) Single confocal sections showing immunofluorescence detection of c-Fos protein expression at various times after SKF 81297 (5.0 mg/kg, i.p.) injection. Scale bar: 40 µm. (B) Quantification of c-Fos immunoreactive neurons in mice treated with vehicle or SKF 81297 (5.0 mg/kg, i.p.) at various time points. Data (means ± SEM; n = 3–6 mice per group) were analyzed using one-way ANOVA: c-Fos (F_(5,12)_ = 6.14; p<0.05). Bonferroni-Dunn test: NS.
(TIF)

Figure S2 **SKF 81297, SKF 83822 and SKF 83959 induce ERK phosphorylation in the dentate gyrus.** (A) Single confocal section showing P-ERK in the DG following systemic administration of SKF 81297 (5.0 mg/kg, i.p.), SKF 83822 (2.0 mg/kg, i.p.) and SKF 83959 (2.0 mg/kg, i.p.). Scale bar: 40 µm. (B) Quantification of P-ERK immunoreactive neurons after pharmacological treatment with the D1-type receptor agonists. Data (means ± SEM; n = 3–4 mice per group) were analyzed using one-way ANOVA: treatment (F_(3,10)_ = 236.16; p<0.001). Bonferroni-Dunn test: *** p<0.001 and °°° p<0.001.
(TIF)
