# Supplementary figures and images for: Convulsant Doses of a Dopamine D1 Receptor Agonist Result in Erk-Dependent Increases in Zif268 and Arc/Arg3.1 Expression in Mouse Dentate Gyrus
Source: PLoS One. 2011 May 3;6(5):e19415. doi: 10.1371/journal.pone.0019415 (PMC3086923; doi:10.1371/journal.pone.0019415)

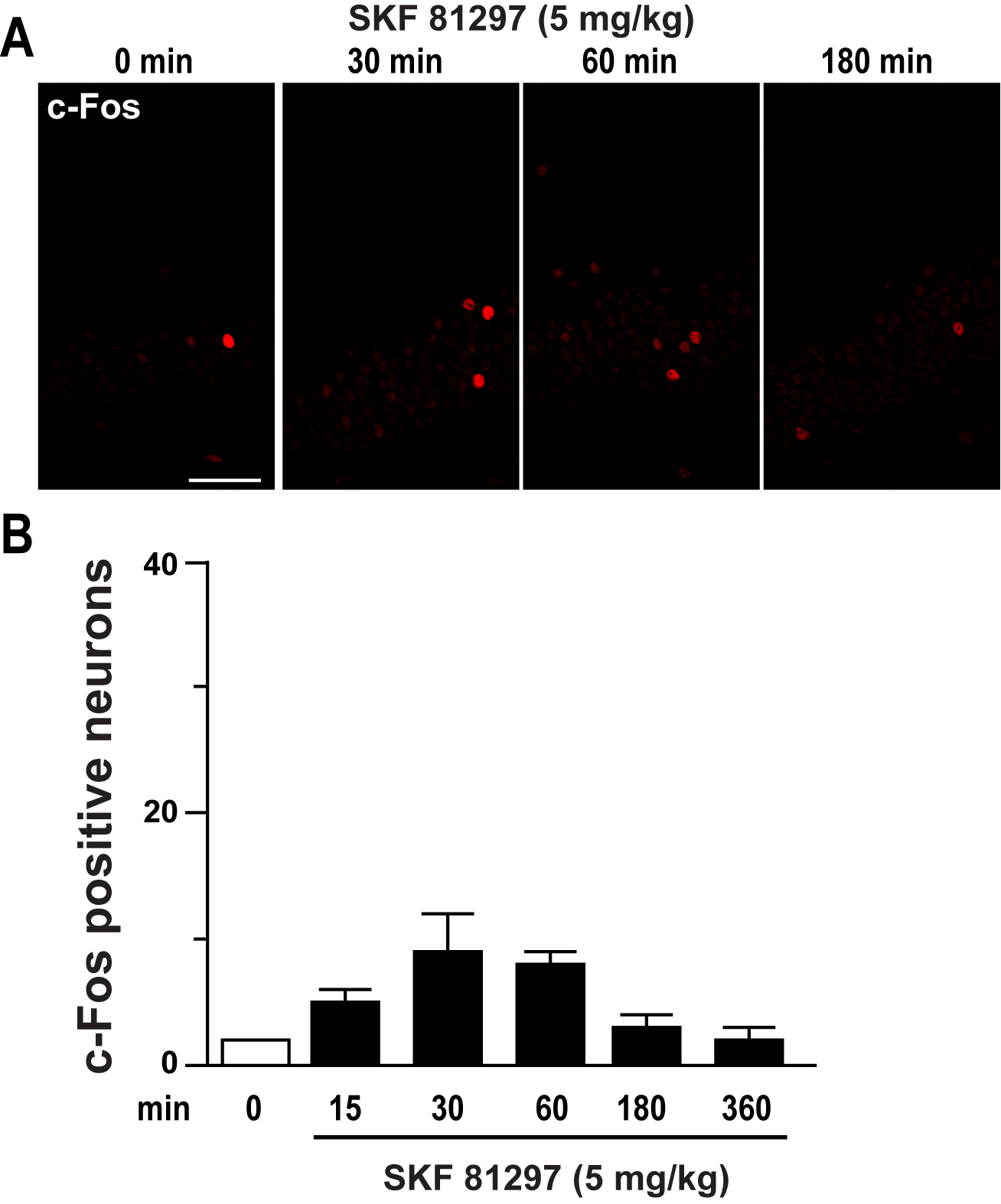

Supplement: Figure S1 — Effect of SKF 81297 on c-Fos expression in the dentate gyrus. (A) Single confocal sections showing immunofluorescence detection of c-Fos protein expression at various times after SKF 81297 (5.0 mg/kg, i.p.) injection. Scale bar: 40 µm. (B) Quantification of c-Fos immunoreactive neurons in mice treated with vehicle or SKF 81297 (5.0 mg/kg, i.p.) at various time points. Data (means ± SEM; n = 3–6 mice per group) were analyzed using one-way ANOVA: c-Fos (F(5,12) = 6.14; p<0.05). Bonferroni-Dunn test: NS. (TIF) [file pone.0019415.s001.tif]

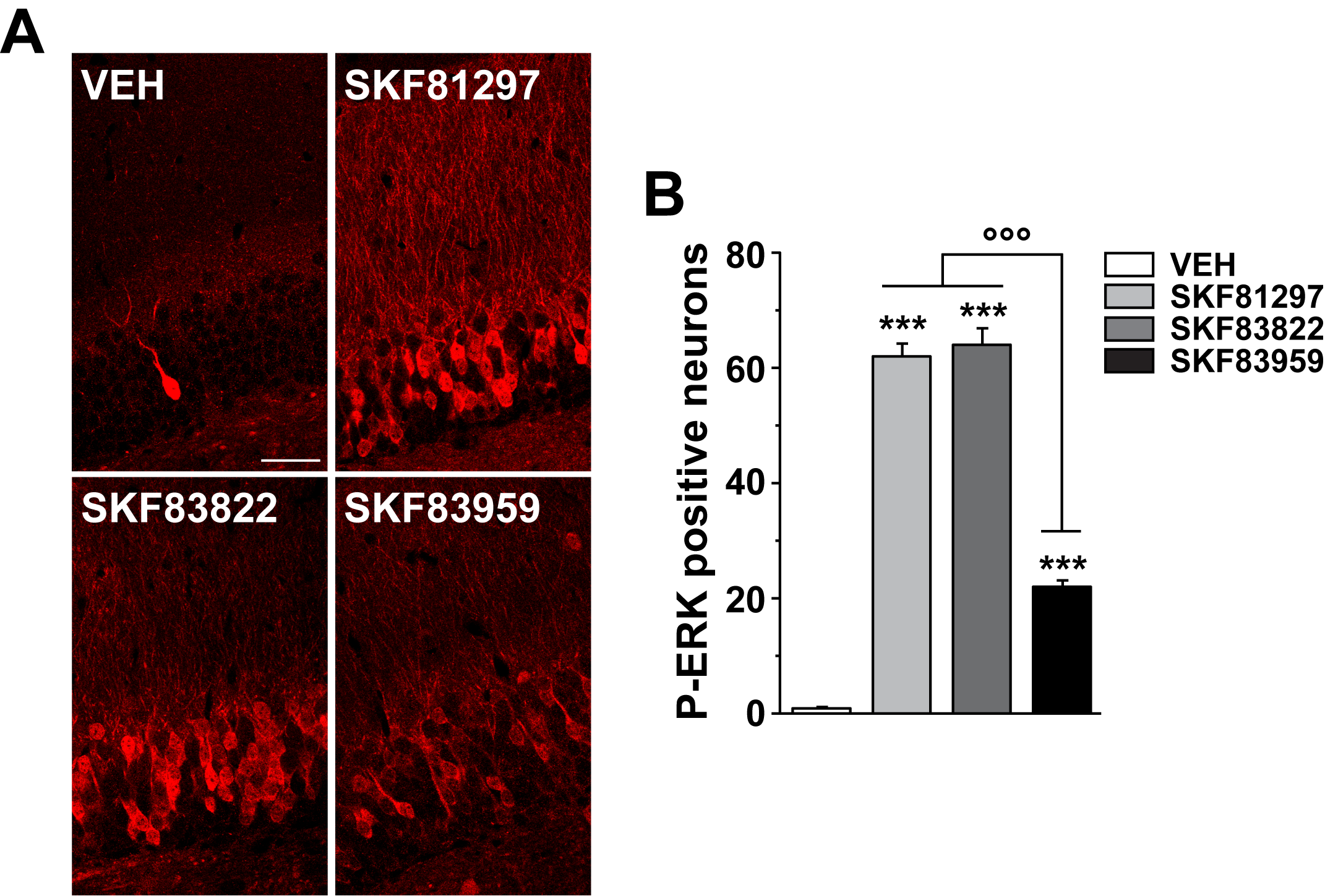

Supplement: Figure S2 — SKF 81297, SKF 83822 and SKF 83959 induce ERK phosphorylation in the dentate gyrus. (A) Single confocal section showing P-ERK in the DG following systemic administration of SKF 81297 (5.0 mg/kg, i.p.), SKF 83822 (2.0 mg/kg, i.p.) and SKF 83959 (2.0 mg/kg, i.p.). Scale bar: 40 µm. (B) Quantification of P-ERK immunoreactive neurons after pharmacological treatment with the D1-type receptor agonists. Data (means ± SEM; n = 3–4 mice per group) were analyzed using one-way ANOVA: treatment (F(3,10) = 236.16; p<0.001). Bonferroni-Dunn test: *** p<0.001 and °°° p<0.001. (TIF) [file pone.0019415.s002.tif]
